# Characterization and Antioxidant Activity of Volatile Constituents from Different Parts of *Aframomum danielli* (Hook) K. Schum

**DOI:** 10.3390/medicines4020029

**Published:** 2017-05-10

**Authors:** Emmanuel E. Essien, Paul S. Thomas, Kelly Oriakhi, Mohammad I. Choudhary

**Affiliations:** 1 Department of Chemistry, University of Uyo, Uyo 520101, Nigeria; emmanuelessien@uniuyo.edu.ng; 2 Hussain Ebrahim Jamal Research Institute of Chemistry, International Centre for Chemical and Biological Sciences, University of Karachi, Karachi 75270, Pakistan; kelly.oriakhi@uniben.edu (K.O.); hej@cyber.net (M.I.C.); 3 Department of Pharmacognosy and Natural Medicine, Faculty of Pharmacy, University of Uyo, Uyo 520101, Nigeria; 4 Department of Medical Biochemistry, University of Benin, Benin 300283, Nigeria

**Keywords:** zingiberaceae, *Aframomum daniell*, essential oils, antioxidant activity, 1,8-cineole, β-pinene

## Abstract

**Background:** *Aframomum danielli* is used in ethno-medicine for the treatment of several ailments and as a traditional food spice. **Methods:** The hydro-distilled leaf, stem, seed, rhizome and pod volatile oils of *A. danielli* were subjected to gas chromatography-mass spectrometry (GC-MS) analysis. Free radical scavenging capacity of the volatile oils was determined using 2,2-diphenyl-1-picrylhydrazyl (DPPH) and ferric reducing antioxidant power (FRAP) assays. **Results:** Thirty-nine (39) volatile compounds were identified in the oils of *A. danielli*, accounting for 85.33 to 96.03% of the total oil composition. The leaf, stem, rhizome and pod volatile oils were dominant in β-pinene (30.94–47.55%), while the seed oil contained a high amount of 1,8-cineole (eucalyptol) (53.44%). The seed oil showed higher radical inhibitory activity in the DPPH assay (IC_50_ value, 45.5 µg/mL) and the rhizome oil was the most effective in the FRAP assay. **Conclusions:** The characterization of the leaf, stem, rhizome and pod volatile oils of *A. danielli* is reported for the first time. *A. danielli* seed and rhizome oils elicit promise as potential plant resource and warrant further biological exploitation.

## 1. Introduction

Spices play a crucial role in nutrition, medicine and fragrance industry. The genus *Aframomum* (family: Zingiberaceae) contains almost fifty species in West and Central Africa [1]. The distinguishing feature of this genus is the attribute of highly pungent and aromatic seeds [2]. All the plant parts also exude a strong aroma when pulverized. *A. danielli* is a perennial plant with seeds used for flavouring traditional dishes, in addition to having laxative, anti-helmintic and anti-fungal properties; juice extract of its rhizomes are effective in the treatment of body odor and toothache [3]. The seed essential oil composition of *A. danielli* from Cameroon, Nigeria and S. Tome has been reported [4,5,6]. Similarly, volatile constituents of other *Aframomum* species grown in some regions of West and East Africa have been investigated [7,8,9,10,11,12,13].

Some biological activities such as antiprotozoal, antibacterial, anti-inflammatory and antioxidant activities have been documented for a number of *Aframomum* species [12,13,14,15,16]. However, there is a paucity of data on the volatile oil composition of other aromatic parts of *Aframomum danielli*, other than the seed oils earlier reported. Moreover, there is an upsurge in the search for natural antioxidants especially of plant origin to protect the human body from free radicals, as well as to hinder the progress of many chronic diseases and of rancidity in food. This work was therefore carried out to determine the composition and antioxidant activity of volatile oils from the seed, leaf, rhizome, stem and pod of *A. danielli*.

## 2. Materials and Methods

### 2.1. Plant Sample

The leaf, stem, seed, rhizome, and pod of *A. danielli* were collected from mature plants cultivated in Uyo Local Government Area of Akwa Ibom State, Nigeria, in the month of March, 2016. The sample was identified by a taxonomist in the Department of Botany and Ecological Studies, University of Uyo, where the voucher specimen was deposited. The essential oils were obtained by hydro-distillation (4 h) of the fresh plant parts using a Clevenger-type apparatus (Garg Process Glass, India Pvt. Ltd., Mumbai, India) in accordance with the British Pharmacopoeia [17]. The oils were dried over sodium sulfate (Sigma-Aldrich, St. Louis, MO, USA) and stored in refrigeration (4 °C) after the estimation of percentage yield.

### 2.2. Gas Chromatography-Mass Spectrometry (GC-MS)

The volatile oils were subjected to GC-MS analysis on an Agilent system consisting of a model 7890N gas chromatograph, a model mass detector Triple Quad 7000A in electron impact (EI) mode at 70 eV (*m*/*z* range 40–600 amu) (Agilent Technologies, Santa Clara, CA, USA), and an Agilent ChemStation data system (Agilent Technologies, Santa Clara, CA, USA). The GC column (Crawford Scientific Ltd, Scotland, UK) was an HP-5ms fused silica capillary with a (5% phenyl)-methyl polysiloxane stationary phase (30 m × 250 μm × 0.25 μm). The carrier gas was helium with a column head pressure of 9.7853 psi and a flow rate of 1.2 mL/min. Inlet temperature and mass selective detector (MDS) temperature was 250 °C. The GC oven temperature program was used as follows: 50 °C initial temperature, held for 5 min; increased at 6 °C/min to 190 °C for 20 min; increased at 7 °C/min to 290 °C for 15 min; increased at 7 °C/min to 300 °C for 10 min. The sample was dissolved in dichloromethane, and 2 µL was injected (split ratio 10:1; split flow 12 mL/min).

The components were identified by comparison of their mass spectra with NIST 1998 library data of the GC-MS system as well as by comparison of their retention indices (RI) with the relevant literature data [18]. The relative amount of each individual component of the essential oil was expressed as the percentage of the peak area relative to the total peak area. The RI value of each component was determined relative to the retention times of a homologous *n*-alkane series with linear interpolation on the HP-5ms column.

### 2.3. Antioxidant Activity

#### 2.3.1. DPPH Radical Scavenging Activity

The DPPH free radical scavenging of the *A. danielli* essential oils and ascorbic acid prepared in methanol at various concentrations (20–100 µg/mL) were evaluated according to the method of Shekhar and Anju [19]. One millilitre of 0.1 mM DPPH solution in methanol was added to 3 mL the solutions prepared with the oils and standard, and stirred for 1 min. Each mixture was kept in the dark at room temperature for 30 min and the absorbance was recorded against a blank at 517 nm. The assays were carried out in triplicate and the results were expressed as mean values ± standard deviation. Lower absorbance of the reaction mixture indicated higher free radical activity. Percentage scavenging activity was calculated using the expression:

(1) $$\%\mathrm{Scavenging}\;\mathrm{activity}=\;\frac{\mathrm{Absorbance}\;\mathrm{of}\;\mathrm{Control}-\mathrm{Absorbance}\;\mathrm{of}\;\mathrm{Sample}}{\mathrm{Absorbance}\;\mathrm{of}\;\mathrm{Control}}\;\times \;100$$

#### 2.3.2. FRAP Assay

The reducing power of the essential oils was determined according to the method of Oyaizu [20]. Various concentrations (20, 40, 60, 80 and 100 µg/mL) of essential oils and ascorbic acid were mixed with phosphate buffer (2.5 mL, 0.2 M, pH 6.6) and 1% (*w*/*v*) of potassium ferricyanide water solution (2.5 mL). The mixture was incubated at 50 °C for 20 min. Aliquots of trichloroacetic acid (2.5 mL, 10%, aqueous solution (*w*/*v*)) were added to the mixture and centrifuged at 3000 rpm for 10 min. The supernatant (2.5 mL) was mixed with distilled water (2.5 mL) and a freshly prepared ferric chloride solution (0.5 mL, 0.1% (*w*/*v*)). After 30 min of incubation at room temperature in the dark, the absorbance of the solution was measured at 700 nm. The experiment was performed in triplicate and the average absorbance was noted for each measurement. Higher absorbance indicated higher reducing power. The ferric-reducing capacity of the essential oils and standard compound were expressed graphically by plotting the absorbance against concentration.

## 3. Results and Discussion

A total of 39 volatile constituents in the leaf, stem, seed, rhizome and pod of *A. danielli* essential oils were identified by GC-MS analysis (Table 1); oil yield of 1.0%, 0.65%, 1.3%, 1.1% and 0.55% were obtained, respectively. The percentage of identified components in the analysis ranged from 85.33 to 96.03% (Table 2). Individual oils displayed their unique chemical profile in quantity and quality characterized by a high amount of monoterpenoids (60.74–89.83%) and a relatively low level of sesquiterpenoids (3.73–30.5%). It was also observed that *A. danielli* volatile oils contain low amounts of oxygenated compounds in the leaf, stem, rhizome and pod (10.41–19.19%), with the exception of the seed oil, which is dominant in oxygenated constituents (72.63%) (Table 2). 

The high content of 1,8-cineole, α-terpineol and β-pinene in the seed oil of *A. danielli* in this study is likewise reported for the fruit oil of *A. danielli* (25.5–34.4%), β-pinene (14.1–15.2%) and α-terpineol (9.9–12.1%) from S. Tome [6]. Similarly, Adegoke et al. [5] recorded 1,8-cineole (59.8%), β-pinene (13.2%) and α-terpineol (9.3%) in high proportions for *A. danielli* seeds from Nigeria; while 1,8-cineole (48.9%) was shown for *A. danielli* seeds from Cameroon [4]. 

**Figure 1:**
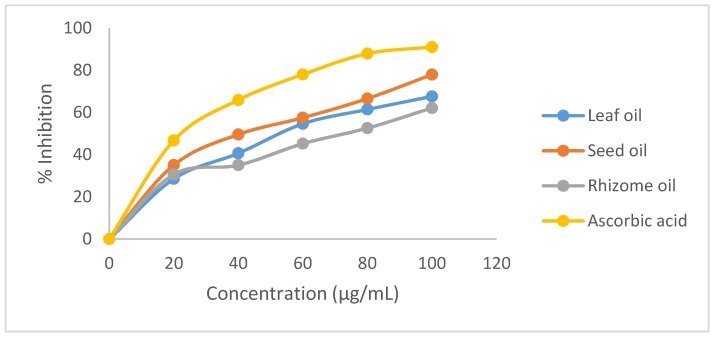
2,2-Dipenyl-1-picrylhydrazyl radical scavenging activity of *A. danielli* essential oils.

The leaf, seeds and rhizome of *A. danielli* are the most frequently used plant portions and are implicated in folkloric claims. Therefore, the antioxidant assays were carried out on these targeted plant parts. Figure 1 is a presentation of the DPPH radical scavenging activity of *A. danielli* leaf, seed and rhizome essential oils compared with the standard compound, ascorbic acid. The plot indicates the scavenging ability of the oils as a percent inhibition at various concentrations; the scavenging effect was concentration dependent. This was demonstrated by the volatile oils’ ability to act as a hydrogen atoms or electrons donor in the conversion of DPPH radical to DPPH-H. The seed oil exhibited the highest percent inhibition in the assay (77.96%, 100 µg/mL) compared with the leaf and rhizome oils (67.59% and 62.11%, respectively, 100 µg/mL), while ascorbic acid showed 91.00% inhibition. DPPH radical activity is usually presented with an IC_50_ value. The IC_50_ values for leaf, seed and rhizome oils were 58.5 µg/mL, 45.5 µg/mL and 72.0 µg/mL, respectively, while ascorbic acid showed a value of 20.5 µg/mL.

Interestingly, the seed oil is shown to be dominant in oxygenated components, such as 1,8-cineole (53.44%) and α-terpineol (12.23%), as compared to the leaf and rhizome oils (Table 1). It is suggestive that 1,8-cinoeole and other oxygenated components of the seed oil may be responsible for the prominent radical scavenging effect; as a consequence, the antioxidant activity depends on individual oil composition. Similarly, α-terpineol, one of the constituents of *Eucalyptus teretecornis* leaf volatile oil, was shown to be responsible for the observed antioxidant effect, though 1,8-cineole, α-pinene and β-pinene occurred as major constituents [21]. Dongmo et al. [16] reported a higher scavenging efficacy of *A. danielli* seed volatile oils (1,8-cineole and linalool as the main oxygenated components) compared to the leaf oil. The leaf and rhizome oils of *A. giganteum* have also been shown to demonstrate good antioxidant effects [8]. 

**Figure 2:**
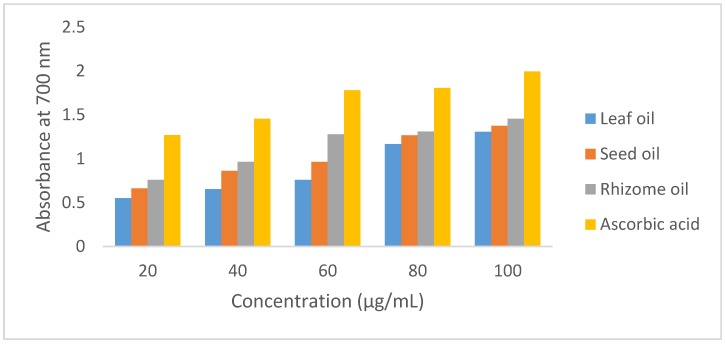
Ferric ion-reducing capacity of *A. danielli* volatile oils.

The concentration-dependent, ferric-reducing potential of *A. danielli* oils and ascorbic acid (as a function of absorbance at 700 nm) is shown in Figure 2. The reducing capacity of the volatile oils and standard compound increased with a corresponding increase in concentration. The FRAP assay indicated the higher reduction ability of the rhizome oil, followed by the seed and leaf oils (1.454, 1.376 and 1.305, respectively, at 100 µg/mL). This is an indication of the *A. danielli* oil constituents’ ability to reduce the (Fe^3+^) to (Fe^2+^) by electron transfer. The ferric ion reduction ability of ascorbic acid in the assay (100 µg/mL, 1.995) was relatively higher than the absorbance values for the volatile oils. The relatively moderate oxygenated proportion of the *A. danielli* rhizome oil may have afforded the observed antioxidant potency in the FRAP assay.

## 4. Conclusions

The seed volatile oil of *A. danielli* comprised mainly of 1,8-cineole and α-terpineol while the leaf, stem, rhizome, and pod oils were dominant in β-pinene. The seed oil exhibited the most prominent antioxidant effect in the DPPH assay, while the rhizome volatile oil was more effective in the FRAP assay; this is an indication of the antioxidant efficacy of the seed and rhizome employed in ethno-medicine. 

## Figures and Tables

**Table 1:** Composition of *A. danielli* essential oils.

Compound	RI	Leaf (%)	Stem (%)	Seed (%)	Rhizome (%)	Pod (%)	QI (%)
**Aliphatic ester**							
2-Pentyl acetate	876	-	-	0.15	-	-	95
**Monoterpenoid hydrocarbons**							
α-Pinene	941	8.9	6.73	2.51	7.15	8.07	98
β-Pinene	982	47.55	30.94	9.15	34.51	34.55	95
β-Myrcene	993	-	1.2	0.39	-	-	94
α-Phellandrene	1006	-	10.12	0.12	8.77	12.26	95
α-Terpinene	1020	-	1.22	0.3	1.2	-	95
p-Cymene	1028	-	6.9	-	-	4.82	95
Limonene	1032	1.59	-	-	-	-	94
β-Ocimene	1052	-	-	0.4	-	-	96
γ-Terpinene	1058	-	8.98	6.42	9.81	13.49	94
α- Terpinolene	1090	-	5.42	0.16	5.25	8.23	96
**Oxygenated monoterpenoids**							
1,8-Cineole	1034	-	-	53.44	1.95	-	98
β-Linalool	1103	-	2.38	0.35	1.61	3.53	97
trans-Pinocarveol	1137	0.57	-	-	-	-	94
Borneol	1168	-	1.69	-	-	-	94
Terpinen-4-ol	1179	0.65	-	0.83	1.25	-	95
α-Terpineol	1191	-	2.7	12.23	4.2	3.98	97
Myrtenol	1195	0.87	-	-	-	-	95
trans-Geraniol	1256	-	-	1.08	-	-	96
α-Bornyl acetate	1287	-	-	-	0.67	-	95
Pinocarvyl acetate	1306	0.61	-	-	-	-	94
Myrtenyl acetate	1327	-	2.03	0.15	1.92	0.9	98
α-Terpineol acetate	1333	-	-	1.06	-	-	94
**Sesquiterpenoid hydrocarbons**							
α-Copaene	1377	-	0.42	1.55	0.38	-	98
α-Cubebene	1352	-	-		0.85	-	95
β-Cubebene	1391	-	-	0.66	-	-	94
α-Gurjunene	1410	-	1.63		3.47	-	96
β-Caryophyllene	1419	9.73	5.29	0.12	-	1.73	98
Alloaromadendrene	1461	-	-	0.36	-	-	93
Germacrene D	1482	-	-	0.47	-	-	94
γ-Cadinene	1513	-	0.33	-	-	-	94
δ-Cadinene	1524	-	-	0.64	-	-	95
α-Caryophyllene	1564	4.28	0.65	-	-	-	97
**Oxygenated sesquiterpenoids**							
Ledol	1566	-	-	0.52	1.16	-	95
trans-Nerolidol	1568	-	0.55	2.67	-	1.35	97
Caryophyllene oxide	1579	14.68	2.1	-	1.18	-	97
Cubenol	1643	-	-	0.3	-	-	96
Longipinocarvone	1639	1.81	-	-	-	-	92
Eudesm-7(11)-en-4-ol	1682	-	-	-	-	0.65	95

RI = experimental retention index on an HP-5ms column; QI = quality index, indicates the fit comparison of the experimental mass spectrum and the NIST library spectrum; - = not detected.

**Table 2:** Percentage composition of classes of compounds in *A. danielli* essential oils.

Compound Class	Leaf (%)	Stem (%)	Seed (%)	Rhizome (%)	Pod (%)
Aliphatic ester	-	-	0.15	-	-
Monoterpenoid hydrocarbons	58.04	71.51	19.6	66.69	81.42
Oxygenated monoterpenoids	2.7	8.8	69.14	11.6	8.41
Sesquiterpenoid hydrocarbons	14.01	8.32	3.8	4.7	1.73
Oxygenated sesquiterpenoids	16.49	2.65	3.49	2.34	2.0
Total monoterpenoids	60.74	80.31	88.74	78.29	89.83
Total sesquiterpenoids	30.50	10.97	7.29	7.04	3.73
Total oxygenated compounds	19.19	11.45	72.63	13.94	10.41
Total identified	91.24	91.28	96.03	85.33	93.56

- = not detected.
